# Sheep Reared on Sewage Sludge–Treated Pasture: Sharpe Responds

**Published:** 2006-02

**Authors:** Richard Sharpe

**Affiliations:** MRC Human Reproductive Sciences Unit, Centre for Reproductive Biology, University of Edinburgh, Edinburgh, United Kingdom, E-mail: r.sharpe@hrsu.mrc.ac.uk

We thank Evans for his interest in our article (Paul et al. 2005) and for his comments. He appears to have misunderstood the purpose of our study, which was to provide evidence as to whether or not the complex mixture of chemicals that are present in treated sewage sludge are able to exert any effects on the developing fetal testis in the sheep. This information might provide insights into current understanding about the impact of environmental chemicals on development and malfunction of the human testis. In this study we chose what we considered to be the most appropriate control treatment, which was to maintain pasture in its normal format according to local environmental conditions with the addition of appropriate amounts of inorganic nitrogenous fertilizer. The aim was not to control exactly for the relative amounts of all other organic and/or inorganic materials because this would be almost impossible to do when considering the complex makeup of sewage sludge.

Possible differential effects of the control and sewage sludge treatment on growth of the sward in the two pastures and their consequent contribution to different nutritional effects in the ewes maintained on that pasture were controlled by varying the stocking levels according to the sward length. This was clearly indicated in the “Materials and Methods” of our article (Paul et al. 2005). Evidence that this approach was successful can be gleaned from the observation that there was no difference in body weight between the pregnant ewes maintained on the two types of pasture. Evans must have misunderstood our article because he seems to think that there was a difference in weight of the pregnant ewes.

Another point raised by Evans is the potential of clovers and their endogenous phytoestrogens to contribute to the changes we observed in our study (Paul et al. 2005). In the pastures in which these studies were conducted (55°N), there were minimal amounts of clover, even after several years of treatment with sludge; therefore, any contribution of this source to our study is almost certainly minimal.

Finally, we point out again that there were no significant differences between the control animals and those reared on sewage sludge–fertilized pastures in terms of the frequency of multiple births, so it is not appropriate to consider Evans’ speculation regarding contributions that the mineral nitrogen might have made to this occurrence.

In summary, although we accept that there may be differences between the two types of pasture that may have contributed in some way to the present studies—for which we have been unable to control—we believe that our study achieved its primary goal: We established that exposure of pregnant ewes to a complex cocktail of environmental chemicals (those present in treated sewage sludge) could selectively affect development of the testes of male fetuses. We have not identified which chemical or mixture of chemicals caused this change, and we emphasized in our article (Paul et al. 2005) that to do so is a complex and probably impossible task. The important point is to prove the principal that exposure to mixtures of environmental chemicals at “real world” levels has the potential to alter male reproductive development.
